# Comprehensive transcriptome characterization of *Grus japonensis* using PacBio SMRT and Illumina sequencing

**DOI:** 10.1038/s41598-021-03474-7

**Published:** 2021-12-14

**Authors:** Wentao Ye, Wei Xu, Nan Xu, Rong Chen, Changhu Lu, Hongyi Liu

**Affiliations:** 1 The Co-Innovation Center for Sustainable Forestry in Southern China, College of Biology and the Environment, Nanjing Forestry University, Nanjing, 210037 China; 2 Nanjing Hongshan Forest Zoo, Nanjing, 210028 China

**Keywords:** Genetics, Zoology

## Abstract

The red-crowned crane (*Grus japonensis*) is an endangered species distributed across southeast Russia, northeast China, Korea, and Japan. Here, we sequenced for the first time the full-length unreferenced transcriptome of red-crowned crane mixed samples using a PacBio Sequel platform. A total of 359,136 circular consensus sequences (CCS) were obtained via clustering to remove redundancy. A total of 303,544 full-length non-chimeric sequences were identified by judging whether CCS contained 5′ and 3′ adapters, and the poly(A) tail. Eight samples were sequenced using Illumina, and PacBio sequencing data were corrected according to the collected Illumina data to obtain more accurate full-length transcripts. A total of 4,100 long non-coding RNAs, 13,115 simple sequences repeat loci and 29 transcription factor families were identified. The expression of lncRNAs and TFs in pancreas was lowest comparing with other tissues. Many enriched immune-related transmission pathways (MHC and IL receptors) were identified in the spleen. This study will contribute to a better understanding of the gene structure and post-transcriptional regulatory network, and provide references for future studies on red-crowned cranes.

## Introduction

The red-crowned crane is a large wading bird that is mainly distributed in the southeast of Russia, northeast of China, Korea, and Japan^1–4^. The population of the red-crowned crane is split into continental and island populations, with continental groups being migratory birds and the island groups being resident birds^5^. In recent years, due to the intensification of human activities and reclaimed wetland, the breeding and wintering habitats of the migratory populations have experienced double loss of function and area^6–8^. Hence, the range of activity of this species in China has been continuously reduced and its population has started to decline significantly, with the number of red-crowned cranes observed in Winter being only 8% of that reported in the 1980s^5^, making it an endangered species, as declared by the International Union for Conservation of Nature in 2012^9^. Despite the awareness that the red-crowned crane is on the brink of extinction and the implementation of several protective measures, such as with the establishment of legislation and key conservation areas, the population of the migratory species continues to decline^2,10,11^. To protect red-crowned crane, many studies about its population dynamics, feeding habits, habitat protection, disease control, overwintering ecology and reproductive behavior have been conducted^7,12–16^, including molecular studies mainly focused on marker development and genetic diversity analysis. Several markers were identified and characterized, such as simple sequence repeats (SSRs) and single nucleotide polymorphisms (SNPs)^11,17,18^ and genetic diversity and population structure of the red-crowned crane populations were estimated using various markers, such as major histocompatibility complex (MHC), SSRs, and mitochondrial genes^19–25^. However, only two studies involving transcriptome and genome of the red-crowned crane have been performed, which included the analysis of the expression profiles of cytochrome P450 (CYP) 1–3 genes in red-crowned crane tissues by Illumina HiSeq 2500 and analysis of the genomic relationships between the crane and other birds by Illumina HiSeq 2000^26,27^.

Although second-generation sequencing is one of the most widely employed sequencing technologies, this is less effective in assembling the full-length transcripts for the limitation of short read length, and thus restricts the yield of full-length genes^28–30^. However, the third-generation sequencing technology represented by Single Molecule Real-Time (SMRT) effectively overcomes this limitation^31^. SMRT sequencing enables direct acquisition of the full-length transcriptome sequence of functional genes with an accuracy as high as 99.999%^32,33^. Thus, SMRT sequencing can be used to identify new functional genes that can supplement the gene information related to important biological process^30,34–36^. Currently, SMRT technology has been used to obtain full-length transcriptional information of several endangered species, such as *Sika deer*, *Manis javanica,* and *Macaca mulatta*^37–39^. Hence, SMRT sequencing technology provides an important method for understanding gene structure and transcription network of endangered animals^34,35^. This is of great significance for studying the regulatory mechanism of endangered animals and the differential expression of environmental impact genes.

In this study, the full-length transcripts of red-crowned crane were obtained for the first time through PacBio SMRT sequencing and the expression landscapes of eight different tissues (brain, muscle, pancreas, heart, kidney, liver, lung, and spleen) were further evaluated using Illumina NovaSeq 6000 sequencing. In addition, detailed transcriptome analysis presented this novel information in light of transcript diversity, comprising information on full-length transcripts, transcription factors (TFs), long non-coding RNAs (lncRNAs), novel genes, SSRs, and immune-related signaling pathways. Therefore, the collected data provides a reference for understanding the gene structure and post-transcriptional regulatory network of red-crowned crane, and may lay the foundation for future molecular research related to red-crowned crane, and contribute to the protection of this endangered species.

## Results and discussion

### High quality non-redundant full-length transcriptome

It has been reported that SMRT Sequel has the advantage of ultra-long reading length, including the 5'- and 3'-untranslated regions (UTRs) and poly(A) tail of the transcript, which provides important information for the stability and translation of mRNA^40^. Herein, we used the PacBio sequence platform to sequence mixed samples of red-crowned crane and obtained a total of 24.54 G subreads base with an average length of 2,335 bp. The subread BAM of the offline data was self-corrected to obtain 359,136 CCS with an average length of 2,779 bp. Moreover, the hierarchical n*log(n) algorithm was used to cluster the full-length non-chimeric sequences of the same transcript to obtain the consensus sequence, which was further polished by arrow software to obtain 23,452 polished consensus. In addition, eight tissue samples (brain, heart, kidney, liver, lung, muscle, pancreas, and spleen) were analyzed by Illumina technology, from which a total of 181,391,092 raw reads were obtained. After filtering the raw data, we obtained 172,306,116 high quality clean reads (Table 1). To more accurately identify the full-length transcriptome, the third-generation data was corrected based on the second-generation data with high accuracy using LoRDEC 0.7 software^41^. Moreover, the CD-HIT software was used to align and cluster the 23,452 polished consensus sequences and remove redundant sequences, providing a total of 15,126 unigenes with an average length of 2,879 bp. The number of genes with different transcript length in the interval (< 0.5, 0.5–1, 1–2, 2–3, and > 3 kb) is shown in Table 2. Overall, 22,994 gene subtypes were identified, with most genes having only one isoform. In addition, 15,126 unigenes obtained after CD-HIT software processing were used as the reference sequence, and the clean reads of each tissue sequenced by Illumina were aligned to the reference sequence using RSEM. We found that the mapping rate between the pancreas and the reference sequence was up to 86.85%, and the minimum mapping rate between the brain and the reference sequence was only 56.36% (Table 3).

**Table 1:** Summary of transcriptome sequencing data obtained using Illumina technology.

Sample	Raw reads	Clean reads	Clean bases (G)	Error (%)	Q20 (%)	Q30 (%)	GC (%)
Lung	24,145,993	22,747,100	6.82	0.03	97.98	94.40	51.03
Heart	21,721,663	20,415,889	6.12	0.03	97.95	94.22	50.12
Liver	22,896,225	21,884,390	6.57	0.03	98.02	94.40	50.89
Spleen	23,934,810	22,751,455	6.83	0.03	97.90	94.22	51.30
Muscle	21,473,933	20,457,834	6.14	0.03	97.84	94.10	53.09
Kidney	23,711,715	22,814,386	6.84	0.03	97.69	93.62	49.21
Brain	22,267,555	21,178,394	6.35	0.03	97.91	94.23	50.50
Pancreas	21,239,198	20,056,668	6.02	0.02	98.39	95.22	55.19

**Table 2:** Comparison of subreads and corrected reads from PacBio sequencing.

Transcripts length interval	< 500 bp	500–1000 bp	1000–2000 bp	2000–3000 bp	> 3000 bp	Total
Number of transcripts	51	678	7,221	7,336	8,166	23,452
Number of Genes	29	391	4,110	4,641	5,955	15,126

**Table 3:** The number of reads from Illumina sequencing assembling to PacBio sequencing.

Sample	Total reads	Mapped reads (percentage)
Brain	42,356,788	23,872,756 (56.36%)
Heart	40,831,778	28,422,308 (69.61%)
Kidney	45,628,772	28,929,056 (63.40%)
Liver	43,768,780	31,899,618 (72.88%)
Lung	45,494,200	26,477,848 (58.20%)
Muscle	40,915,668	27,774,902 (67.88%)
Pancreas	40,113,336	34,839,298 (86.85%)
Spleen	45,502,910	25,910,924 (56.94%)

Compared with the traditional second-generation sequencing, the PacBio SMRT technology used in this study significantly improved the reading ability, not only providing the full-length transcripts, but also identifying lncRNAs, SSRs, and TF families. Regardless of whether it was a coding or non-coding gene, the prevalence of long transcripts was higher than previously predicted. We could predict the gene function of the obtained long transcripts and obtain more accurate information on red-crowned crane interleukin (IL) and MHC receptor families, which can provide additional insights into the immune function. Our sequencing research has largely filled the gap in the red-crowned crane transcriptome and provided a reference for discovering new protein coding genes and transcripts.

### Functional annotation of the transcripts

**Figure 1:**
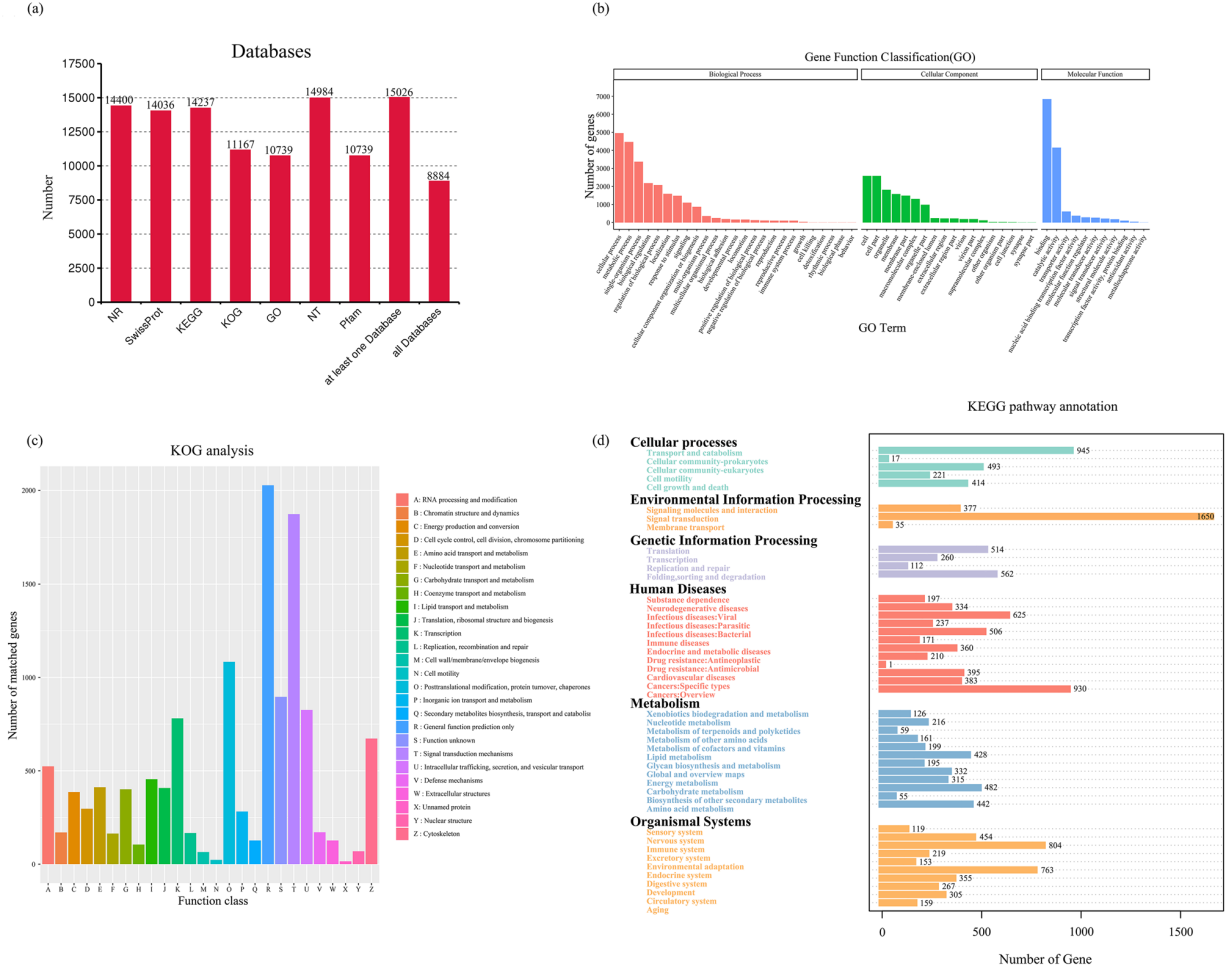
Functional annotation of the unigenes identified in different samples of red-crowned crane. (**a**) Number of transcripts annotated by BLASTx against the available databases. (**b**) GO functional annotations according to “cellular components”, “biological processes”, and “molecular functions”. The abscissa shows gene functions and the ordinate the number of transcripts with GO functions. (**c**) KOG functional annotations. The abscissa shows the function class and the ordinate is the number of matched genes. (**d**) KEGG pathway annotations. The abscissa shows the number of genes and the ordinate the gene functions.

To obtain the most abundant and complete annotation information, we annotated all sequences based on the sequence similarity search of seven major databases, including National Center for Biotechnology Information (NCBI) non-redundant proteins (NR), NCBI nucleotide sequences (NT), Protein family (Pfam), euKaryotic Ortholog Groups (KOG), a manually annotated and reviewed protein sequence database (Swiss-Prot), Kyoto Encyclopedia of Genes and Genomes (KEGG), and Gene Ontology (GO). A total of 15,026 (99.34%) unigenes were successfully matched to known sequences or domains in at least one of the seven databases, of which 8,884 (58.73%) unigenes were annotated in all databases. In addition, 14,400 (95.20%), 14,036 (92.79%), 14,237 (94.12%), 11,167 (73.83%), 10,739 (70.99%), 14,984 (99.06%) unigenes were annotated in NR, Swiss-Prot, KEGG, KOG, GO, NT, and Pfam database, respectively (Fig. 1a). To further elucidate the main biological functions of red-crowned crane unigenes, GO, KOG, and KEGG pathways analyses were performed. A total of 10,739 unigenes were annotated using the GO database, which were further divided into 54 major functional groups according to biological processes, cellular component, and molecular function. We found that the main subgroups of biological processes were "cellular process" (GO: 0,009,987, 4,951) and "metabolic process" (GO: 0,008,152, 4,465). In the classification of cellular components, the predominant portion of transcripts represented “cell” (GO: 0,005,623, 2,584) and "cell part" (GO: 0,044,464, 2,587). The main categories in the classification of molecular functions were "binding" (GO: 0,005,488, 6840) and "catalytic activity" (GO: 0,003,824, 4149) (Fig. 1b). In addition, a total of 11,167 unigenes were subdivided into KOGs, among which "General function prediction only" (2,028, 16.18%) was the largest group, followed by "signal transduction mechanisms" (1,875, 14.96%), "posttranslational modification, protein turnover, chaperones" (1,084, 8.65%) (Fig. 1c). To explore the biological functions and interactions of the identified genes, we searched 34,350 red-crowned crane genes in the KEGG database. A total of 14,237 unigenes were found to match within the database information, and they were assigned to KEGG pathways, which were divided into six sub-categories, i.e., Cellular Processes (2,090), Environmental Information Processing (2,062), Genetic Information Processing (1,448), Human Diseases (4,349), Metabolism (3,010), and Organismal Systems (3,598) (Fig. 1d). These pathways in the KEGG pathway were closely related to normal life activities. “Human Diseases” pathway was annotated by enriched analysis, with the largest group of unigenes being signal transduction belonging to Human Diseases. It should be noted that the samples herein analyzed were collected from a dead red-crowned crane; thus, the main signal pathway of red-crowned cranes at the time of death may be mainly related to " Human Diseases ".

### Analysis of other genetic features

**Figure 2:**
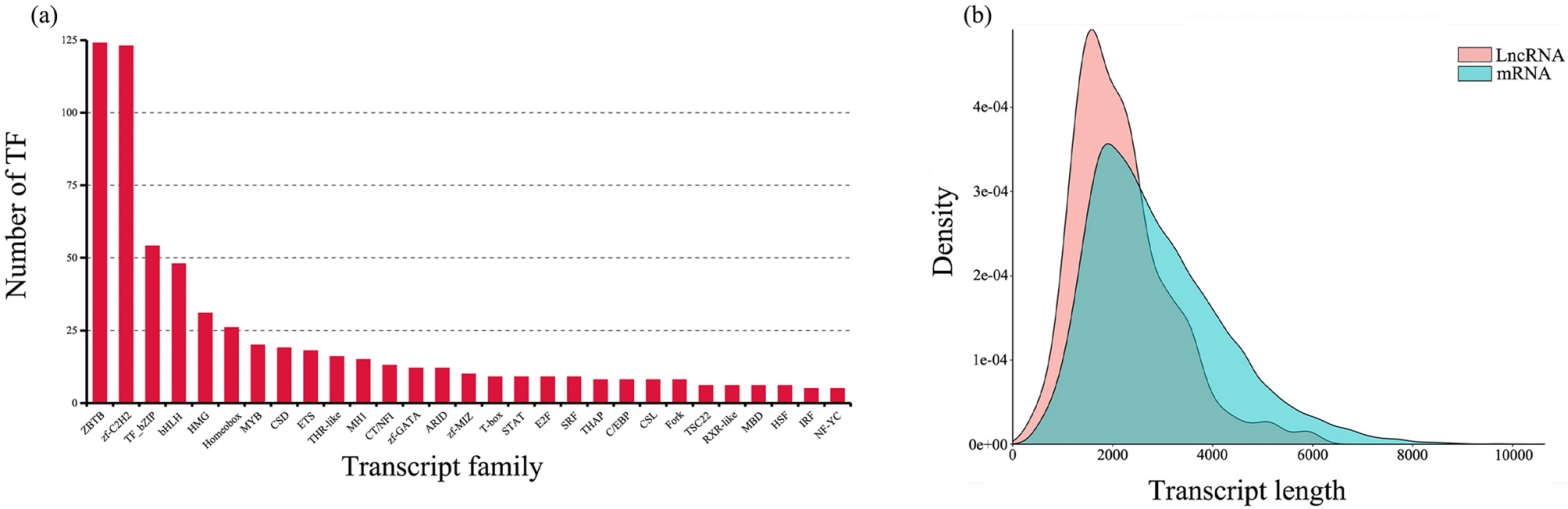
(**a**) Transcription factor analysis results. The ordinate represents the number of transcript factors, and the abscissa the different TF families. (**b**) Annotated lncRNA and mRNA were used to generate length distribution density maps. The ordinate represents the number of transcripts, and the abscissa is the length of the transcripts.

TFs were identified using animalTFDB 2.0 and a total of 29 families were identified (Fig. 2a). ZBTB (124) was the most abundant, followed by zf-C2H2 (123). According to previous studies, TFs of these TF families can directly bind to DNA and control gene expression nearby, thereby regulating gene transcription and playing an important role in biological processes, such as cell proliferation, differentiation, apoptosis, and functional regulation^42,43^. Therefore, the highest expression level of these TF families may be explained by the origin of the samples used in this study, specifically dead red-crowned cranes. In addition, the expression of TFs in the pancreas was lowest, and differed sharply from other tissues (Fig. S1).

LncRNAs play an important role in epigenetics, transcriptional, and post-transcriptional regulation of gene expression. Increased expression of lncRNAs has been found to play an important role in cell physiological activities, as well as in cancer and other diseases. In this study, we obtained 4,100 lncRNAs by coding prediction using CNCI, CPC, Pfam, and PLEK databases, with an average length lower than that of mRNA sequences (Fig. 2b). Furthermore, the expression of lncRNAs was similar to TFs. The expression of lncRNAs in pancreas was lowest, and differed obviously from other tissues (Fig. S2). The herein provided updated high-quality transcriptome of the red-crowned crane contributes to enhanced gene annotation and lncRNA analysis of this species.

Furthermore, a total of 13,115 SSRs loci were identified by PacBio sequencing, the single nucleotide repeat motif was the dominant type, accounting for 76.35% of the total SSRs, followed by two and three nucleotide repeat motifs, with frequencies of 7.05% and 14.27%, respectively (Table 4). The information regarding the SSR loci provides more abundant molecular markers for genetic diversity analysis and map construction of red-crowned crane. These sequencing results make the transcript dataset of red-crowned cranes more complete, which is conducive to providing a reference basis for accurate annotation research of the red-crowned crane in the future.

**Table 4:** Distribution of SSRs with different motif types and repeat numbers in the red-crowned crane.

Motif length	Repeat number					Total	Ratio (%)
	5–8	9–12	13–16	17–20	21–24		
1	0	6157	2867	800	189	10,013	76.35
2	797	112	14	2	0	925	7.05
3	1793	66	6	6	0	1,871	14.27
4	175	2	4	2	0	183	1.40
5	71	9	9	5	0	94	0.71
6	21	4	3	1	0	29	0.22
Total	2857	6350	2903	816	189	13,115	–

### Differential gene expression in different tissues

**Figure 3:**
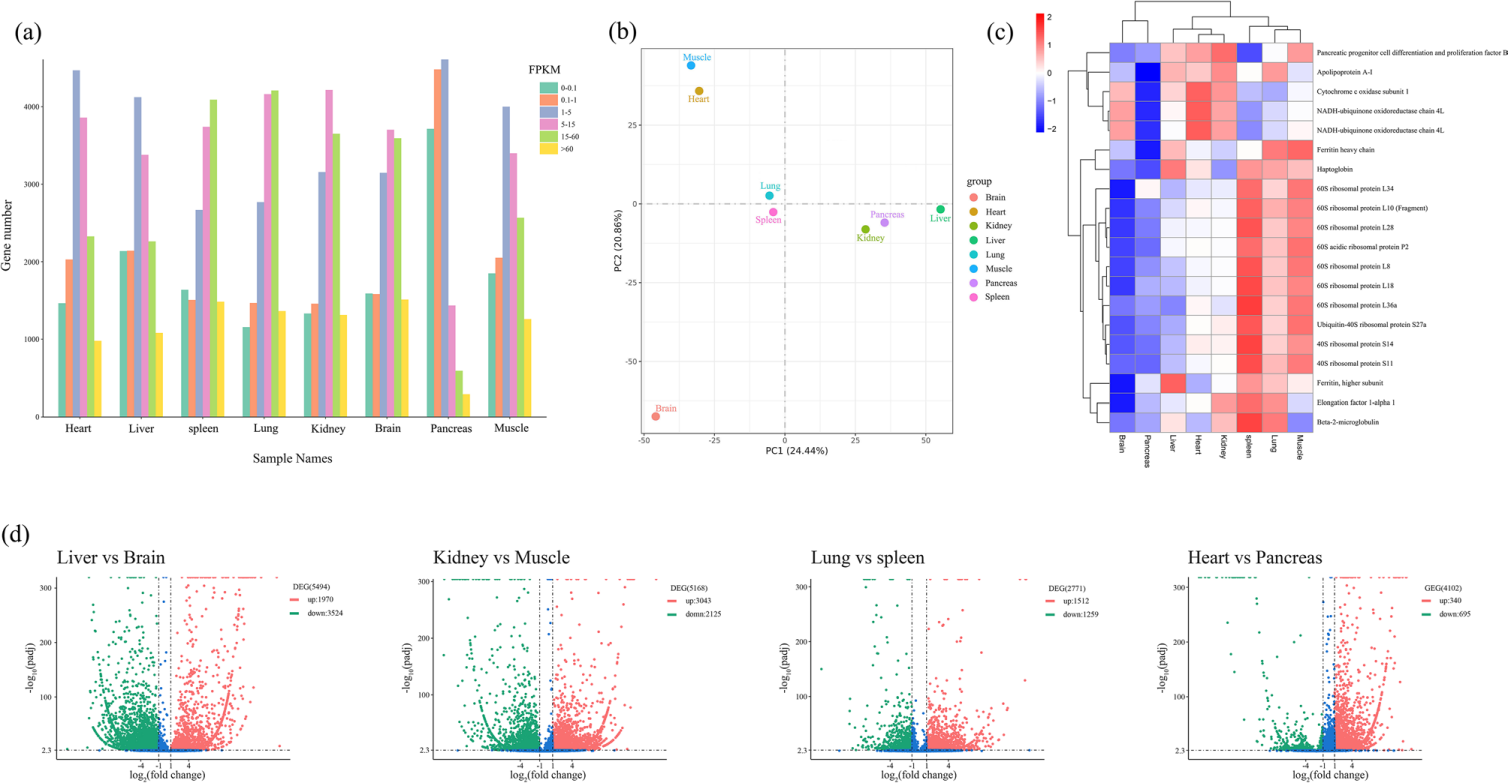
(**a**) Number of genes under different expression conditions in different tissues. (**b**) PCA analysis of the full-length transcriptome. (**c**) Heatmap of the top 20 DEGs within each tissue sample. (**d**) Differential gene expression analysis volcano graph. Red and green dots indicate significantly upregulated and downregulated genes, respectively.

Next, the gene expression profile of different tissues of the red-crowned crane was analyzed and compared. The CD-HIT software was used to remove the redundancy of the corrected consensus sequence, and the unigenes were obtained as the reference sequence. Then, the clean reads of each sample sequenced by Illumina were aligned to the reference sequence by using the comparison software bowtie2 in RSEM, with the highest mapping rate of pancrease being 86.85% (Table 3). Based on gene expression criteria of FPKM > 0.1. We counted the number of genes under different expression conditions and found that the genes with higher expression were the least in pancrease. This may indicate that the gene expression profile of the pancreas is less affected by external factors (Fig. 3a).

Principal component analysis (PCA) of the full-length transcriptome of eight different tissues of red-crowned crane revealed that the difference between the lung and spleen was the smallest, whereas that between the liver and brain was the largest (Fig. 3b). We analyzed the differential gene expression of the top 20 genes in the different tissues and found that the pancreas had the lowest expression profile (Fig. 3c). To obtain more accurate data, we analyzed the expression patterns of 17,535 genes and drew a volcano map comparing differential gene expression between two tissues. The largest difference was between the liver and the brain with 5,494 differentially expressed genes (DEGs), of which 1,970 were upregulated and 3,524 were downregulated. The smallest difference was between the lung and spleen with 2,771 DEGs, of which 1,512 were upregulated and 1,259 were downregulated. Random comparison of the remaining tissues showed 4,102 DEGs between the heart and pancreas, of which 3,407 were upregulated and 695 were downregulated. Moreover, a total of 5,168 DEGs were identified between the kidneys and muscles, including 3,043 upregulated and 2,125 downregulated sequences (Fig. 3d).

### Expression of immune-related IL and MHC receptors

**Figure 4:**
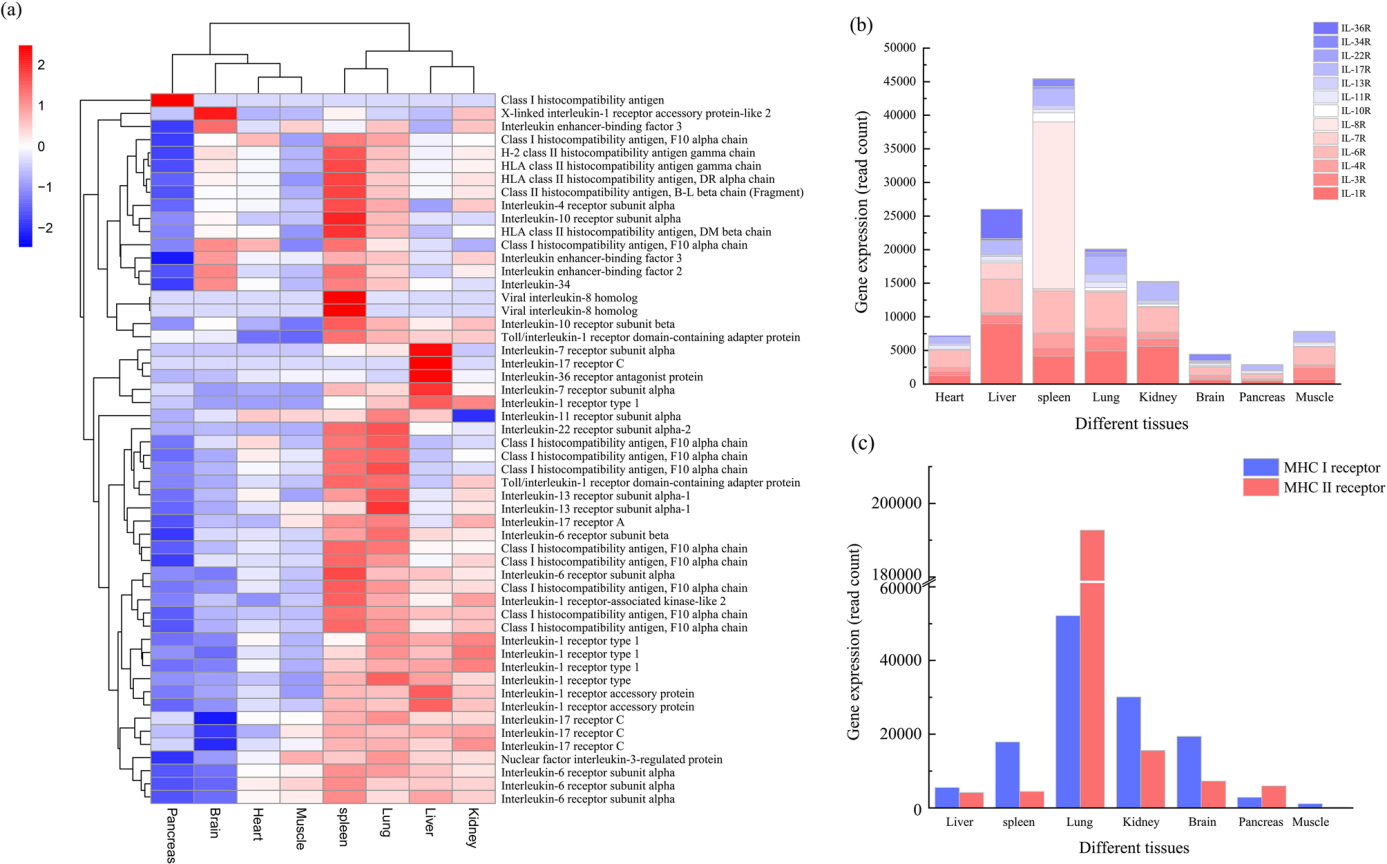
(**a**) Expression of immune related genes analyzed by heatmap. (**b**) Expression of IL receptor family in different tissues. (**c**) Expression of MHC receptor family in different tissues.

To obtain relevant information about the immune system of the red-crowned crane in its infancy, we used the Swiss-Prot database to predict the function of the full-length transcriptome landscape identified for the different tissues. The expression of immune-related genes was analyzed by heatmap, revealing that the spleen was the tissue with the most immune expression (Fig. 4a). Moreover, many IL and MHC receptor families were identified, indicating the innate and adaptive immunity^44^. Among them, 13 types of IL receptors (IL-1R, IL-3R, IL-4R, IL-6R, IL-7R, IL-8R, IL-10R, IL-11R, IL-13R, IL-17R, IL-22R, IL-34R, and IL-36R) and two MHC receptors (MHC I and MHC II) were identified. The IL receptor family was expressed mostly in the spleen, whereas was the lowest in the pancreas. In the spleen, the expression of IL-8R was the highest and that of IL-36R was the lowest (Fig. 4b). The MHC I and MHCII receptors family was expressed mostly in the lung, whereas was the lowest in the muscle. The number of MHC II receptors in the spleen was much higher than that of MHC I receptors, as the spleen is more sensitive to exogenous antigens (Fig. 4c). Through the expression of IL and MHC receptor families in different tissues, we identified that the spleen was the main immune organ in the red-crowned crane that died due to a leg bone fracture. As red-crowned cranes are endangered wild animals, we only use random individuals that died of disease for analysis; thus, we could not repeat our experiments to further confirm the obtained results. Most full-length transcripts were found to be enriched in immune-related signaling pathways, as expected in a sick state^30,36,45–47^. Accordingly, the immune expression of the spleen was found to be the highest, suggesting that the spleen is the main immunity organ of the red-crowned cranes beyond all doubt.

## Conclusion

In summary, we reported the first full-length transcriptome for critically endangered *Grus japonensis* using PacBio SMRT and Illumina sequencing technology. In this study, we used full-length transcriptome sequencing to present a new set of transcriptomic data comprising 13,115 SSRs, 4,100 lncRNAs, and 29 TFs. The purpose of our research was to obtain a more complete and accurate transcriptome dataset of red-crowned crane through three-generation sequencing technology, and then discuss the relevant tissues involved in immunity, to provide a basis for the research and protection of this endangered species. Through functional annotation and structural analysis of the transcriptome, many enriched immune-related signaling pathways were found. Our sequencing data of the full-length transcriptome of a juvenile red-crowned crane provides valuable information to close the knowledge gap in the transcriptome for this species, thereby providing a reference on new protein-coding genes and transcript subtypes. In conclusion, this study provides enhanced transcriptome information, improves our understanding regarding the gene structure and post-transcriptional regulatory network, and provides a reference for future studies on red-crowned cranes.

## Methods

### Sample collection

**Figure 5:**
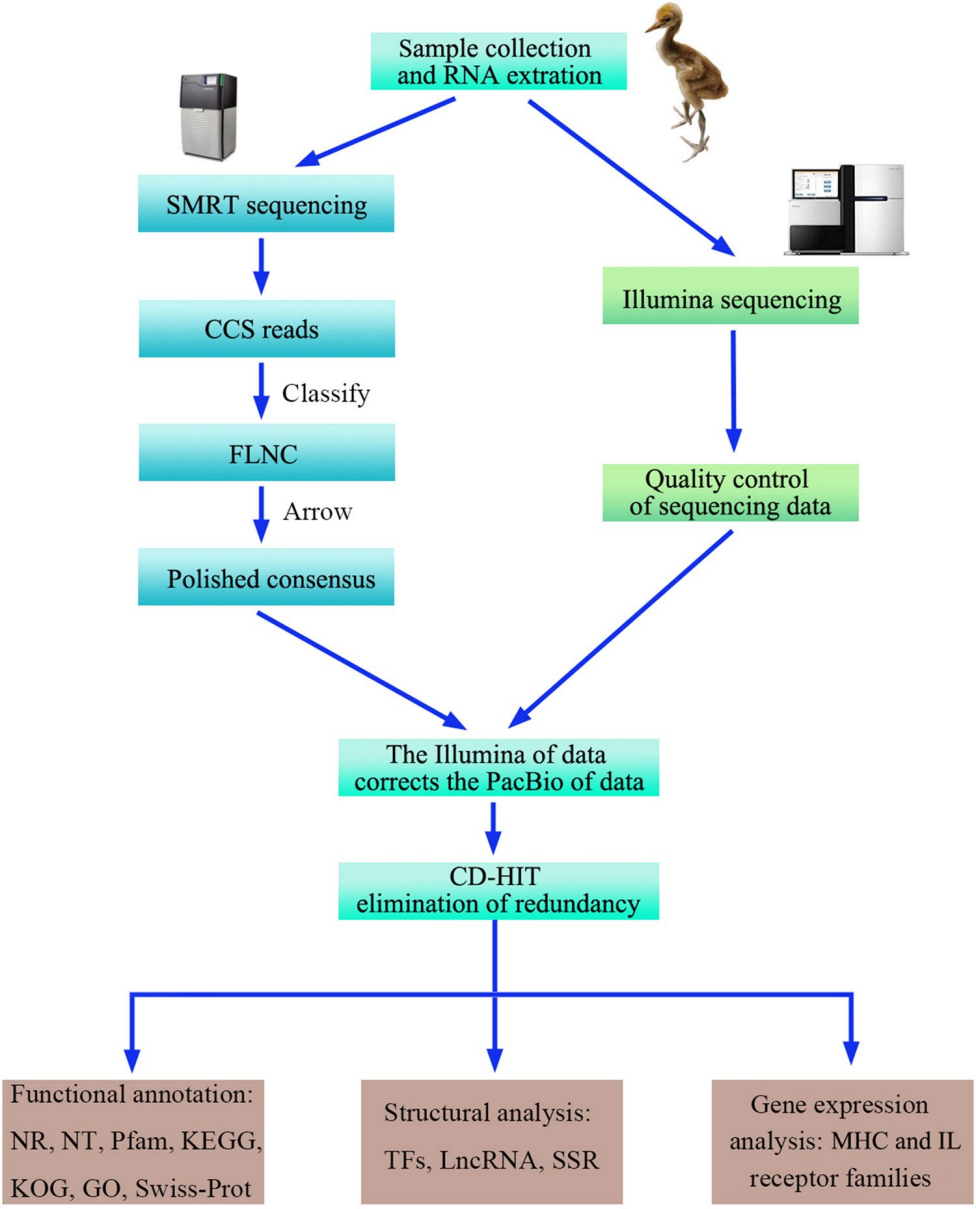
Overview of the experimental design of this study.

The samples used in this study were collected from a red-crowned crane that died due to a leg bone fracture in the Hongshan Forest Zoo of Nanjing, and related scientific research was conducted with the relevant permission from the zoo. To maintain the original state of the samples, they were quickly stored at -80 °C. Brain, muscle, pancreas, heart, kidney, liver, lung, and spleen tissue samples were collected. PacBio SMRT was used to sequence the full-length transcriptome of a mixed sample, and each of the eight tissues were sequenced separately using Illumina sequencing platform NovaSeq 6000. The overall experimental flow chart was drawn by Adobe Photoshop v22.0.0 and shown in (Fig. 5). The project was approved by the Animal Ethics Committee of Nanjing Forestry University and the Nanjing Hongshan Forest Zoo.

### RNA extraction and quality analysis

Trizol reagent was used to obtain total RNA from samples. To ensure that the database data of library construction was of high quality, RNA purity was detected by NanoPhotometer spectrophotometer (Implen, Westlake Village, CA, USA). The Qubit 2.0 Fluorometer (Life Technologies, Waltham, MA, USA) was used to accurately quantify the RNA concentration. The RNA Nano 6000 Assay Kit of the Bioanalyzer 2100 system (Agilent Technologies, Santa Clara, CA, USA) was used to accurately investigate RNA integrity. The results showed that RNA samples could be used for library construction.

### Transcriptome sequencing library construction

mRNA samples were purified using magnetic beads with Oligo (dT), the first cDNA was synthesized by reverse transcription of mRNA using the SMARTer PCR cDNA synthesis Kit (Pacific Biosciences, Menlo Park, CA, USA). The synthesized cDNA was enriched by PCR amplification, the amplified cDNA fragments were divided into different size fractions using the Blue Pippin size selection system (Pacific Biosciences). After obtaining suitable fragments, an additional PCR was performed to obtain enough cDNA. Then, full-length cDNA for damage repair, end repair, and connection of SMRT dumbbell joints. Finally, exonuclease digestion was performed. The library was qualified and sequenced using PacBio Sequel platform.

### Data processing

We used PacBio Sequel to sequence the mixed samples. The PacBio official software package SMRTlink 7.0 (Parameter: minLength = 50, maxLength = 15,000, minpasses 1) was used to process the original Iso-Seq offline data. CCSs were generated from subread BAM files (parameters: min_length, 50; max_drop_fraction, 0.8; no_polish, TRUE; min_zscore, − 9999.0; min_passes, 1; min_predicted_accuracy, 0.8; max_length, 15,000)^46,48^. According to sequence, whether it contained 5 ' and 3' adapters and poly(A) tail, the sequence was divided into full-length or non-full length sequences. The hierarchical n*log(n) algorithm was used to cluster the full-length sequences and obtain a cluster consensus sequence, and then the final arrow polishing (parameters: hq_quiver_min_accuracy, 0.99; bin_by_primer, false; bin_size_kb, 1; qv_trim_5p, 100; qv_trim_3p, 30) was performed^46,49^. The obtained consensus sequences were calibrated with the non-full-length sequences to obtain high-quality sequences for subsequent analysis. The LoRDEC 0.7 software was used to correct with high accuracy the third-generation data based on the second-generation data^41^. Then, CD-HIT (-c 0.95-T 6-G 0-aL 0.00-aS 0.99) software was used for sequence alignment clustering. Redundant sequences were removed based on 95% similarity.

### Gene functional annotation

The final transcript data were evaluated using seven different databases (NCBI NR, NCBI NT, Pfam (http://pfam.sanger.ac.uk/), KOG (http://www.ncbi.nlm.nih.gov/COG/), Swiss-Prot (http://www.ebi.ac.uk/uniprot/), KEGG (https://www.genome.jp/kegg/), and GO (http://www.geneontology.org/)) to obtain comprehensive functional gene information. SSRs were identified using MISA 1.0 (http://pgrc.ipk-gatersleben.de/misa/misa.html). TFs were predicted using the AnimalTFDB 2.0 database (http://bioinfo.life.hust.edu.cn/AnimalTFDB/). Highly credible lncRNA were obtained through PLEK (https://sourceforge.net/projects/plek/) using the k-mer scheme to predict potential coding capabilities. CNCI (https://github.com/www-bioinfo-org/CNCI) was based on coding and non-coding sequence analysis to obtain more accurate lncRNA. CPC2 (http://cpc2.cbi.pku.edu.cn/download.php) was used to predict potential coding capabilities. PfamScan database (https://www.ebi.ac.uk/seqdb/confluence/display/THD/PfamScan) was used to obtain final lncRNA sequence by hmmscan homologous.

### Differential expression analysis

The corrected consensus sequence was further filtered using CD-HIT, and the obtained unigenes were used as the reference sequence. The clean reads of each tissue sequenced by Illumina were aligned to the reference sequence using RSEM v1.3.0 (–phred33; -quals; –forward-prob 0.5; –time)^50^. Gene expression was estimated using RSEM for the eight tissue samples obtained from *Grus japonensis*. The expression profiles of the unigenes in different cDNA libraries were detected in terms of fragments per kilobase of transcript per million mapped reads (FPKM). We used RSEM software to calculate the mapping results, and further obtain the read counts value of each sample mapped to each gene and perform FPKM conversion. The standard of gene expression was set as FPKM > 0.1. R software was used for performing the PCA of the full-length transcriptome. The top 20 DEGs among the eight tissues were screened by coefficient of variation, using NovoMagic v3.0 to draw the heatmaps. In addition, we also did cluster analyses on the expression of lncRNAs and TFs in different tissues (log 2 transformation for IncRNAs and log 10 transformation for TFs).

## Supplementary Information

Supplementary Information.

## Data Availability

We have deposited the primary data underlying these analyses as follows: SubmissionID: SUB8474518; BioProject ID: PRJNA674955; BioSample accessions: SAMN16684236, SAMN16684237, SAMN16684238, SAMN16684239, SAMN16684240, SAMN16684241, SAMN16684242, SAMN16684243, SAMN16684244. All project information will be accessible in the following link: http://www.ncbi.nlm. nih.gov/bioproject/674,955.
