# Supplementary material for: Comprehensive transcriptome characterization of Grus japonensis using PacBio SMRT and Illumina sequencing
Source: Sci Rep. 2021 Dec 14;11:23927. doi: 10.1038/s41598-021-03474-7 (PMC8671462; doi:10.1038/s41598-021-03474-7)
Supplement: Supplementary file 1 — Supplementary Information. [file 41598_2021_3474_MOESM1_ESM.docx]

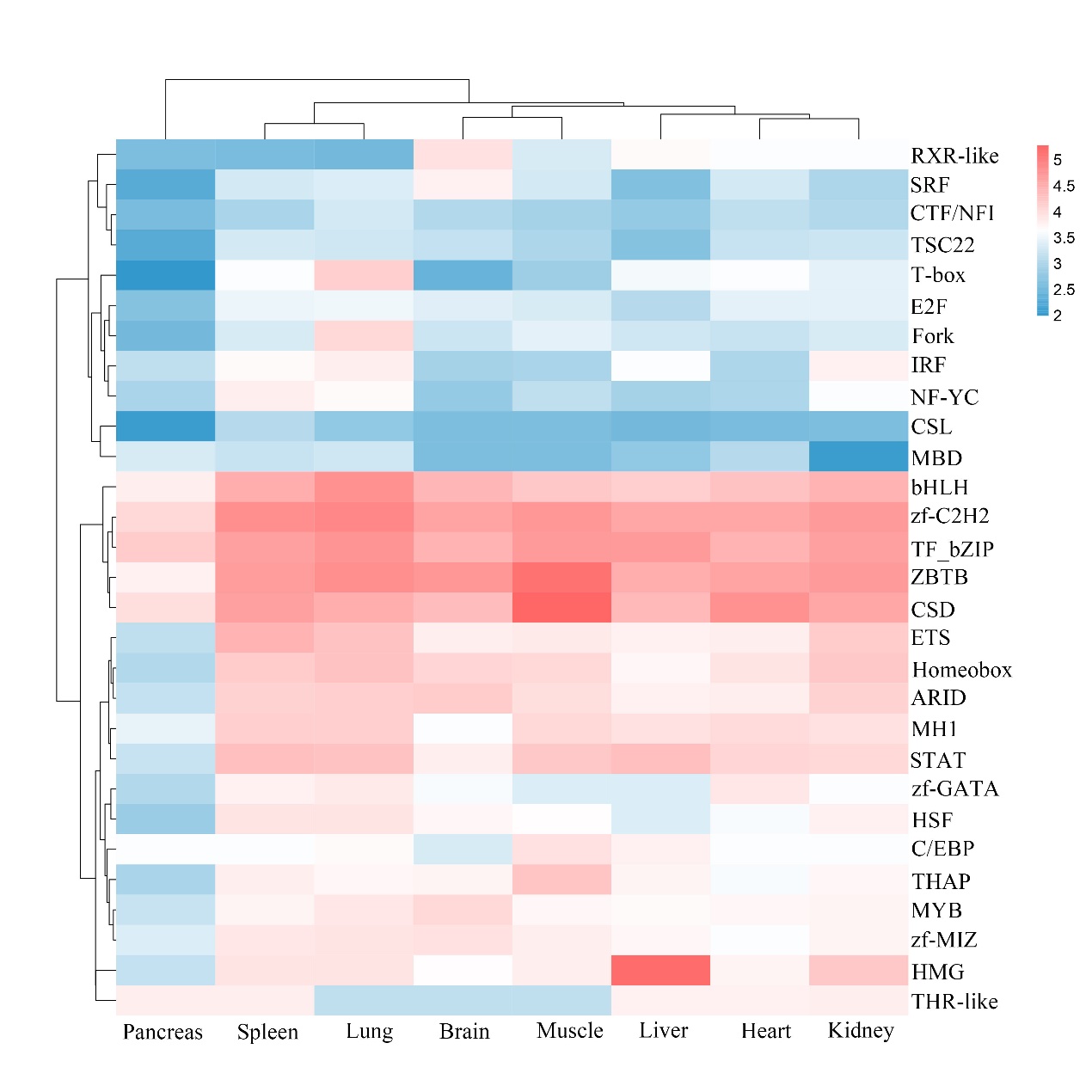


**Fig S1** Expression of TFs in different tissues


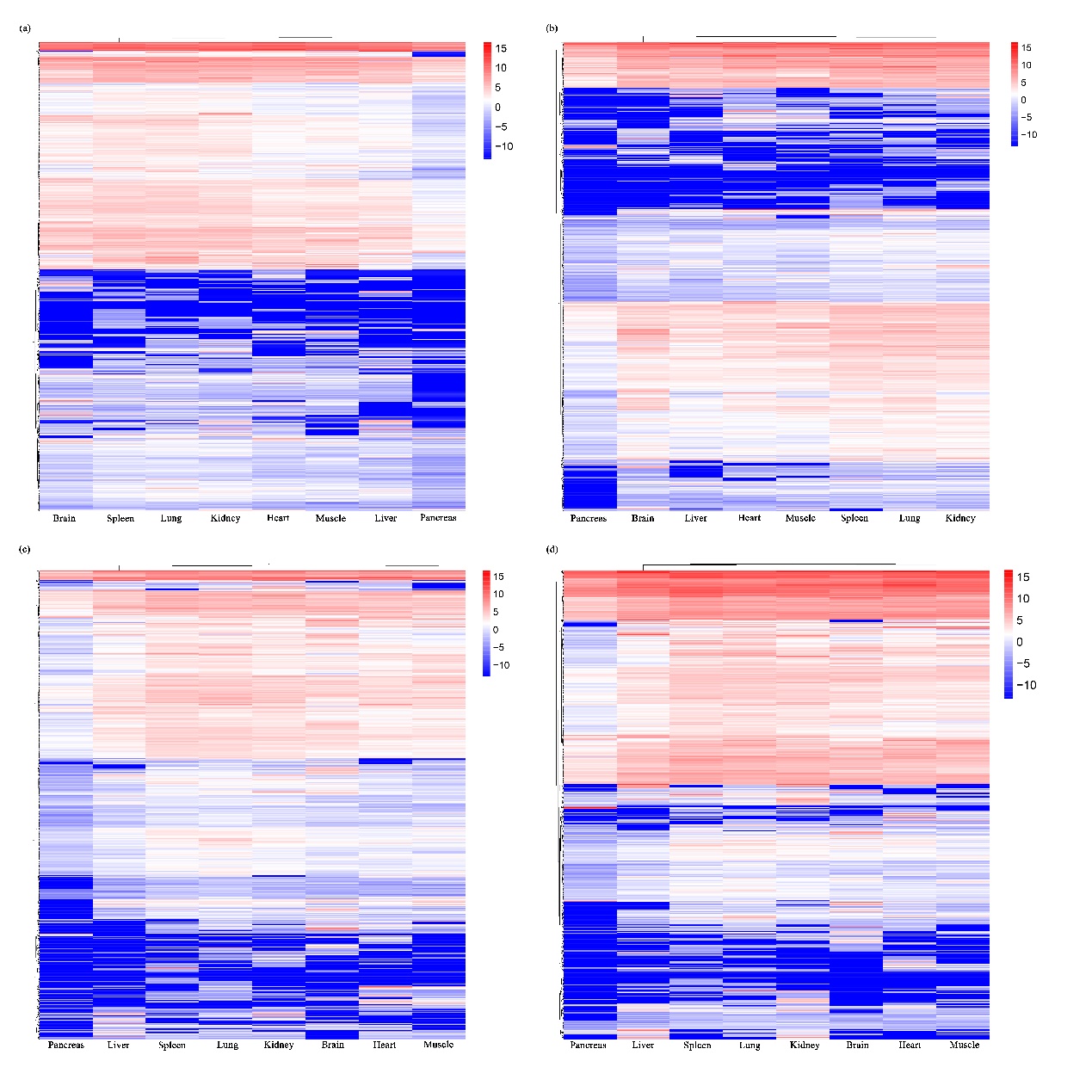


**Fig S2** Expression of lncRNAs in different tissues based on CNCI (a), CPC (b), PFAM (c), and PLEK (d)
